# Heavy Metal Pollution Delineation Based on Uncertainty in a Coastal Industrial City in the Yangtze River Delta, China

**DOI:** 10.3390/ijerph15040710

**Published:** 2018-04-10

**Authors:** Bifeng Hu, Ruiying Zhao, Songchao Chen, Yue Zhou, Bin Jin, Yan Li, Zhou Shi

**Affiliations:** 1 Institute of Agricultural Remote Sensing and Information Technology Application, Zhejiang University, Hangzhou 310058, China; hubifeng@zju.edu.cn (B.H.); ruiyingzhao@zju.edu.cn (R.Z.); yuezhou@zju.edu.cn (Y.Z.); shizhou@zju.edu.cn (Z.S.); 2 Unité de Recherche en Science du Sol, INRA, Orléans 45075, France; 3 InfoSol, INRA, US 1106, Orléans F-4075, France; songchao.chen@inra.fr; 4 Sciences de la Terre et de l’Univers, Orléans University, Orleans 45067, France; 5 Unité Mixte de Rercherche (UMR) Sol Agro et hydrosystème Spatialisation (SAS), INRA, Agrocampus Ouest, Rennes 35042, France; 6 Ningbo Agricultural Food Safety Management Station, Ningbo 315000, China; dd923068626@163.com; 7 Institute of Land Science and Property, School of Public Affairs, Zhejiang University, Hangzhou 310058, China

**Keywords:** soil heavy metal pollution, indicator kriging (IK), Nemerow integrated pollution index (NIPI), uncertainty, pollution area definition

## Abstract

Assessing heavy metal pollution and delineating pollution are the bases for evaluating pollution and determining a cost-effective remediation plan. Most existing studies are based on the spatial distribution of pollutants but ignore related uncertainty. In this study, eight heavy-metal concentrations (Cr, Pb, Cd, Hg, Zn, Cu, Ni, and Zn) were collected at 1040 sampling sites in a coastal industrial city in the Yangtze River Delta, China. The single pollution index (PI) and Nemerow integrated pollution index (NIPI) were calculated for every surface sample (0–20 cm) to assess the degree of heavy metal pollution. Ordinary kriging (OK) was used to map the spatial distribution of heavy metals content and NIPI. Then, we delineated composite heavy metal contamination based on the uncertainty produced by indicator kriging (IK). The results showed that mean values of all PIs and NIPIs were at safe levels. Heavy metals were most accumulated in the central portion of the study area. Based on IK, the spatial probability of composite heavy metal pollution was computed. The probability of composite contamination in the central core urban area was highest. A probability of 0.6 was found as the optimum probability threshold to delineate polluted areas from unpolluted areas for integrative heavy metal contamination. Results of pollution delineation based on uncertainty showed the proportion of false negative error areas was 6.34%, while the proportion of false positive error areas was 0.86%. The accuracy of the classification was 92.80%. This indicated the method we developed is a valuable tool for delineating heavy metal pollution.

## 1. Introduction

Anthropogenic activities, such as urbanization processes, not only change the inherent properties of affected soils, such as their pH, texture, cation exchange capacity, and bulk density, but also inadvertently cause harmful substances, such as heavy metals, to deposit into the soils [1,2,3]. Heavy metals are natural components of the earth’s crust, and natural concentrations of heavy metals in soils tend to remain low. However, anthropogenic inputs of several heavy metals into soils greatly exceed natural inputs from pedogenesis [4]. This problem is more prominent in cities, since cities have a high density of anthropogenic activities and populations. Therefore, citizens in cities face greater health threats from heavy metal soil pollution. In China, the Cr, Pb, Cd, Hg, As, Cu, Zn, and Ni concentrations of soils have frequently been found to exceed the national standard value [5,6,7]. It has been revealed that 16.1% of soil has been polluted by heavy metals according to a national survey of soil pollution released in 2014 by the Ministry of Land and Resources and the Ministry of Environmental Protection of the People’s Republic of China [8]. Heavy metal pollution in soil attracts great attention, since it can have a substantially harmful effect on human health by the ways of consumption of infected animals, and the chronic low-level intake of soil metals through ingestion or inhalation [9,10].

Recently, soils have been widely used as a diagnostic tool to determine environmental conditions that influence health [11,12]. However, there is a need for better information on heavy metal contamination in urban soils [13,14,15,16,17]). Accurately delineating pollution is the aim of pollution studies, and is vital to determining cost-effective remediation plans [18]. In addition, most of the existing related studies are based on the spatial distribution of pollutants [19,20,21,22,23,24,25,26,27], which is usually determined through a number of geostatistical interpolation techniques (generally termed kriging) [28], such as simple kriging (SK) [29], ordinary kriging (OK) [30], cokriging [31], and universal kriging [32]. However, due to frequent, very strong positive skewness of pollution data, the use of these methods is not always optimal and results in uncertainty in the estimations. As a simple but useful method, indicator kriging (IK) has been widely used to map the probabilities of estimates that exceed given threshold values, such as standard values of heavy metal contamination in soil [33,34,35]. To estimate the probability that the heavy metal concentrations exceed the critical threshold, we need the entire probability distributions of heavy metal contents at points. We may assume that the estimation errors follow normal distributions, but this is unrealistic in many situations. Other solutions are to use disjunctive kriging [36] or indicator kriging [37]. Since indicator kriging can accommodate measurements less than the detection limit [38], in this study, a method based on an uncertainty analysis, which was produced by IK, was employed to delineate composite heavy metal pollution contamination. 

The aims of this study were to (1) assess the pollution status of the concentrations of eight heavy metals (Cr, Pb, Cd, Hg, As, Cu, Zn and Ni) in surface soils; (2) investigate the spatial distribution of heavy metals in the surface soils in study area; and (3) explore spatial distribution of uncertainty of composite heavy metals pollution and then delineate pollution sites based on this.

## 2. Methods and Materials

### 2.1. Description of the Case Study Area

The studied area (28°51′–30°33′ N, 120°55′–122°16′ E) is located in the Yangtze River Delta (YRD), which is the most developed economic district in China. It covers an area of 9816 km^2^ and has a population of 7.81 million. It is an important and typical coastal industrial city and is one of 14 cities that implemented the reform and opening policy early in 1984. After that, this area has been undergoing rapid and intense industrialization and urbanization over the past three decades. Nowadays, it is well-known for its industries; chemical, textile and garment, petrochemical engineering, and machinery, and has become an important chemical industrial base in China.

### 2.2. Sampling, Processing, and Analysis

**Figure 1:**
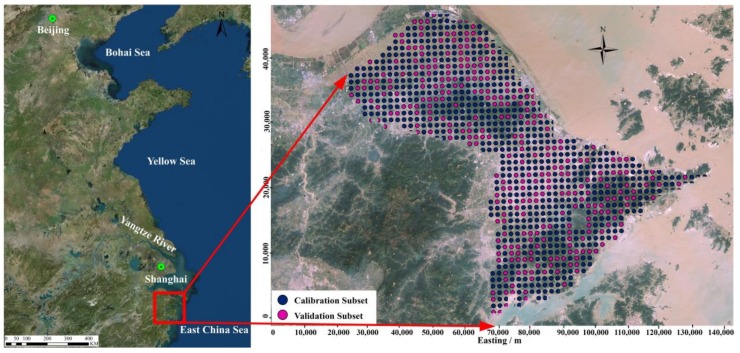
Map of the study area.

Sampling was done in the study area based on 2 × 2 km^2^ grids. In total, 1040 top soil samples (0–20 cm) were collected. A stainless steel hand auger was used. Each soil sample was collected at an intersection point and mixed; five subsamples were collected from five locations within 5 m. A differential global positioning system (GPS) was used to record spatial position of sampling locations (Figure 1). The details of soil and plant sample analysis have been described in previous study [10].

### 2.3. Assessment of Heavy Metal Pollution in Soils

The degree of soil heavy metal pollution was assessed according to related national regulations [39], and a pollution index was used to assess the quality of soil and to estimate the impact of anthropogenic activities [40]. The details are as follows: first, soil pH values were categorized into three classes, <6.5, 6.5 ≤ pH ≤ 7.5, and >7.5; second, the pollution threshold for each soil heavy metal was determined by land use (e.g., paddy fields) and pH class; third, the pollution index (PI) for each heavy metal was determined (Equation (1)); and finally, the Nemerow integrated pollution index (NIPI) was calculated (Equation (2)) [41,42,43,44].

(1) $$PI=C_{i}/S_{i}$$

where $C_{i}$ is the concentration of the soil heavy metal *i* and *S_i_* is the pollution threshold of i heavy metal i in soil.

(2) $$NIPI=((P_{imax}{)}^{2}+{\left(\bar{PI}\right)}^{2}{)}^{1/2}$$

where $P_{i\max}$ is the maximum *PI* value of each heavy metal and $\bar{P}$ is the mean *PI* of each heavy metal. NIPI is a comprehensive index which was used to classify the soils in terms of heavy metal pollution. 

PI is divided into four levels from no to high pollution, to indicate the pollution degree and classified as follows: unpolluted (*PI* ≤ 1), slightly polluted (1 < *PI* ≤ 2), moderately polluted (2 < *PI* ≤ 3), and highly polluted (*PI* > 3). However, the classification of *NIPI* is slightly different from the *PI* levels, and can be graded as safe (*NIPI* ≤ 0.7), precaution (0.7 < *NIPI* ≤ 1.0), slight pollution (1.0 < *NIPI* ≤ 2.0), moderate pollution (2.0< *NIPI* ≤ 3.0), and heavy pollution (*NIPI* > 3.0)

### 2.4. Spatial Distribution of Heavy Metals in Soil

Spatial variability in the concentrations of heavy metals was determined using geostatistical methods. Experimental semivariograms were developed to reveal the spatial dependence of soil properties, using the following equation [45]:

(3) $$\gamma^{*}(h)=\frac{1}{2N(h)}\sum_{i=1}^{N(h)}{\left[Z(x_{i})-Z(x_{i}+h)\right]}^{2}$$

where $\gamma^{*}(h)$ is the semivariance, N(h) is the number of experimental pairs separated by distance h, $Z(x_{i})$ is the measured sample value at point I, and $Z(x_{i}+h)$ is the measured sample value at point i+h. From the analysis of the experimental variogram, a suitable model is typically fit using weighted least squares and parameters such as range, nugget, and sill prior to the kriging procedure. All geostatistical analyses were carried out using ArcGIS 10.2 (ESRI, ArcGIS 10.2, Redlands, CA, USA). Maps of the spatial distribution of heavy metal concentrations in the study area were generated using ordinary kriging (OK) interpolation of the data in the surface and subsurface soils.

### 2.5. Delineating Soil Heavy Metal Pollution Based on Uncertainty Analysis

The sample was divided as calibration subset and validation subset with a ratio of 2:1 with a calibration subset of 693 samples and a validation subset of 347 samples. OK and IK was employed on calibration subset to get the spatial distribution of NIPI and spatial map of probability of NIPI > 1. IK is a kriging analysis performed on a binary-transformed sample population. This analysis considers the problem of estimating the probability that the concentration of a pollutant Z exceeds a critical threshold $Z_{c}$ at an unsampled point u. This approach that was first proposed by Journel (1983) [46] can be used if the spatial correlation of a highly variant parameter is difficult to describe with the raw data. Defining indicators for variables would lead to the following transformation [45]:

(4) $$I(x;z)=\{\begin{array}{cc} 1 & Z(x)\geq z\\ 0 & Z(x)<z \end{array}$$

After transforming the observed data to a new set of indicator variables, the experimental semivariogram is calculated for every set of indicators at each cutoff $Z_{k}$ as

(5) $$\gamma_{1}^{*}(h)=\frac{1}{2N(h)}\sum_{i=1}^{N(h)}{\left[I(x_{i};Z_{k})-I(x_{i}+h;Z_{k})\right]}^{2}$$

where $\gamma_{1}^{*}(h)$ is the indicator experimental semivariogram and N(h) is the number of pairs of indicator transformations $I(\chi_{i};Z_{k})$ and $I(\chi_{i}+h;Z_{k})$ separated by distance vector h.

The conditional cumulative distribution function (ccdf) at each unsampled location, e.g., $\chi_{0}$, is then obtained by the indicator kriging estimator:

(6) $$F(\chi_{0};Z_{k}|(n))=I^{*}(\chi_{0};Z_{k})=\sum_{i=1}^{n}\lambda_{i}I(\chi_{i};Z_{k})$$

where $I^{*}(\chi_{0};Z_{k})$ is the estimated indicator transformation at the unsampled location $\chi_{0}$ and $\lambda_{i}$ is the weight assigned to the indicator transformation I at location $x_{i}$. 

The main steps of delineating heavy metal soil pollution in this study are as follows:

(1) *IK* was employed on calibration subset to evaluate the spatial distribution of the probability of *NIPI* > 1.0, which is the probability of composite heavy metal pollution in the study region. The higher the pollution probability is, the less the uncertainty is. Therefore, we have a sufficient basis to delineate the area with a high pollution probability as the contaminated zone.
(2) To obtain the optimal probability for delineating pollution, misclassifications of samples in validation subset as contaminated or clean with different pollution probabilities were plotted. The probability that had the highest accuracy was selected as the optimal threshold probability, meaning that a location with a pollution probability larger than this threshold was regarded as contaminated land; otherwise, the site was classified as clean land.
(3) The pollution area was delineated according to the optimal pollution probability.
(4) Misclassification rates of delineating pollution based on composited heavy metal pollution uncertainty based on IK and spatial distribution of NIPI through OK were calculated and compared using a validation subset. Misclassification includes false positive errors, which classifies uncontaminated samples as contaminated sites, resulting in unnecessary expenditure on site remediation, and false negative errors, which classify polluted sites as unpolluted sites, leading to a potential decline in human health.

### 2.6. Data Analysis

Microsoft Excel 2010 (Office 2010, Redmond, WA, USA) was deployed to make statistical analyses, and ArcGIS10.2 software (ESRI, ArcGIS 10.2, Redlands, CA, USA) and GS 9.0+ (Gamma Design Software, GS + 9.0, LLC Plainwell, MI, USA) were used to map the sampling sites and perform geostatistical analyses.

## 3. Results and Discussion

### 3.1. Exploratory Data Analysis

The mean contents of As, Cd, Cr, Hg, Ni, Pb, and Zn were 6.55, 0.19, 61.84, 33.87, 0.27, 23.85, 39.86, and 99.60 mg/kg, respectively. The mean concentrations of all heavy metals in the samples were less than the mean concentrations in the national secondary standard (CEPA, 1995) [47]. However, the highest levels of Cd, Cu, Hg, Ni, Cu, and Zn were higher than the concentrations in the national secondary standard. The coefficients of variation for heavy metal concentrations decreased in the following order: Cd > Hg > Cu > Pb > Ni > Zn > Cr > As. Among them, As, Cr, Ni, Pb, Cu and Zn exhibited moderate variability, with coefficients of variation of 34.61%, 38.68%, 46.61%, 47.59%, 92.99% and 40.77%, respectively, and Cd and Hg exhibited the greatest variability, with coefficients of variation of 195.13% and 121.82% (as shown in Table 1).

### 3.2. Heavy Metal Pollution Assessment

The mean PIs of studied heavy metals Cr, Pb, Hg, Cd, AS, Cu, Zn and Ni in soil were 0.24, 0.13, 0.54, 0.31, 0.24, 0.33, 0.40 and 0.48, respectively (Table 2). The mean PI of all the heavy metals in the samples was less than 1, which indicated the content of the heavy metals in soil in study are were at safe levels. However, it is important to note that the maximum PIs for Cr and Pb signified that these metals were at low pollution levels; based on the maximum PI for Ni and Zn, these metals were at mild pollution levels; the maximum PI of Hg, Cu, and Cd indicated that they were at a severe level of pollution.

The mean value of the NIPI was 0.59, which was a safe level. However, the maximum NIPI was 14.02, and this level indicates a severe level of pollution. Overall, the soil contamination in the study area was at levels considered safe.

### 3.3. Spatial Distribution of Soil Heavy Metals

The semivariograms for the four heavy metals are listed in Table 3. The semivariogram of Cr, Hg, As, Cu, and Ni were well fitted with the spherical model, while the semivariogram of Pb, Cd, and Zn were well fitted with the exponential model. The nugget/sill ratio can be used to classify the spatial dependence of heavy metals. If the ratio is less than 25%, then the variable has strong spatial dependence. When the ratio is between 25% and 75%, the variable has moderate spatial dependence, while the variable shows weak spatial dependence when the ratio is greater than 75% [49]. 

The nugget/sill ratios ranged from 26.0% to 49.8% for Pb, Cu, Zn, As and Cd, which indicates that these metals all had moderate spatial dependence. This result indicated that the spatial variation of Pb, Cu, Zn, As, and Cd were affected by both structural and random factors. The nugget/sill ratios for Ni, Cr, and Hg ranged from 10.6% to 17.7%, which indicates that these metals all have strong spatial dependence. This result revealed that the spatial variation of Ni, Cr, and Hg are mainly controlled by structural factors.

**Figure 2:**
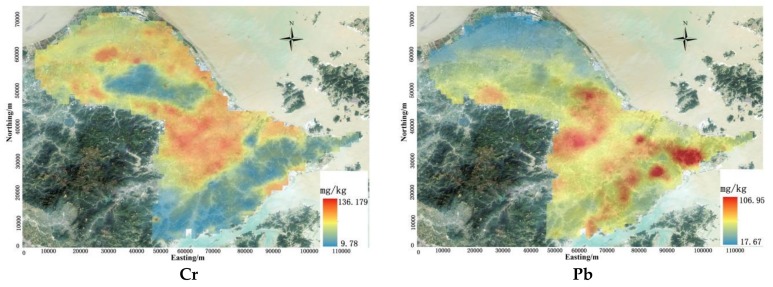

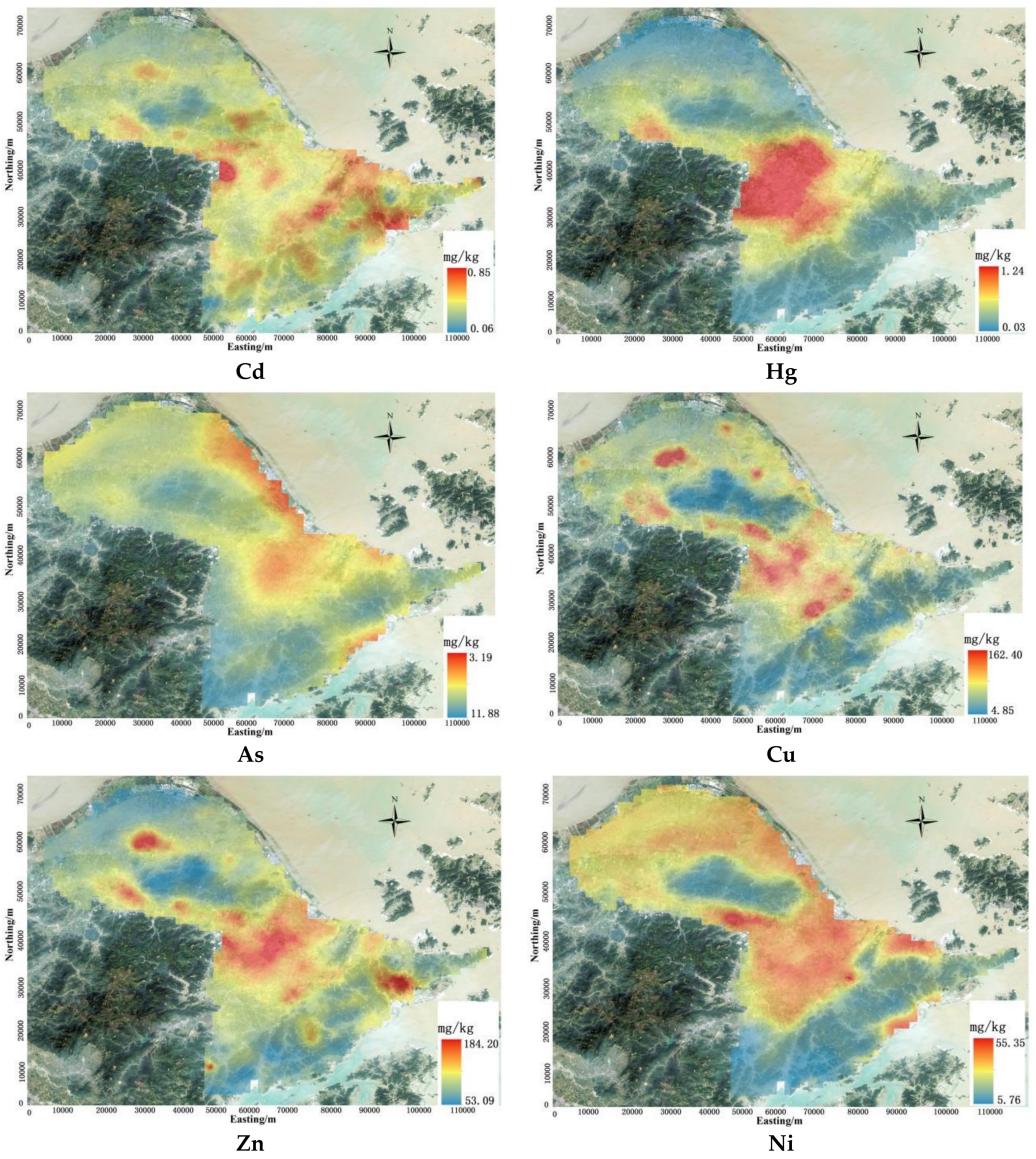
Spatial distribution of heavy metal concentrations.

The highest Cr concentration in the surface soil was in the central portion of the study area. In the southern part of the study area, the Cr content was very low. The Pb content was high in the central and southern portions of the study area and low in the other areas. The Cd value was high in the mid-western and southeastern parts of the study area, while the value was low mainly in the northern portion of the study area. The concentration of Hg in the central part of the study area was higher than in any other places in the study area. The As and Ni concentrations were low in the southern and central portions of the study area and high in other parts of the study area. The concentrations of Cu and Zn were high in the central region and low in other areas (Figure 2). The central region was the core urban area of the study area. In addition, many anthropogenic sources of metals such as industrial and business production activities, vehicle exhaust and aerial deposition are clustered in the urban area and contribute to the enrichment of heavy metals in the soil.

### 3.4. Delineating Heavy Metal Soil Pollution Based on Uncertainty Analysis

**Figure 3:**
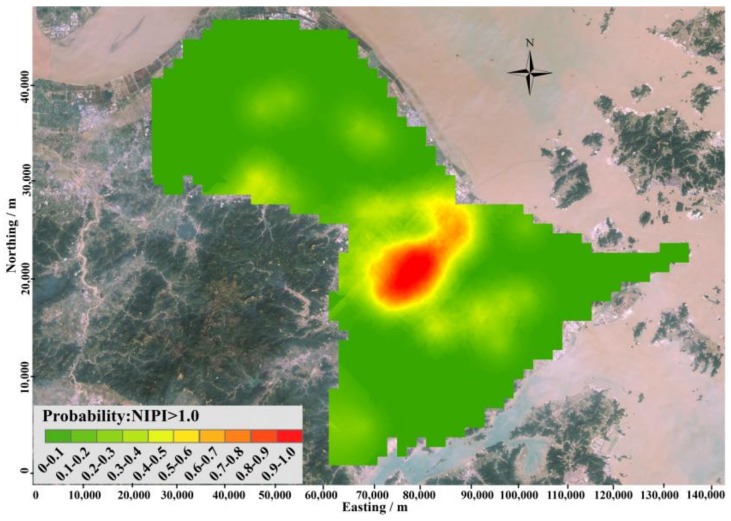
Spatial distribution of the probability of Nemerow integrated pollution index (NIPI) > 1.0.

The spatial distribution of the probability composited heavy metal pollution in the study area is shown in Figure 3. 

As shown in Figure 3, the central portion of the study area had the highest probability of composite heavy metal pollution. The probability of composite heavy metal contamination in the soil in this region was greater than 80%. This result indicated that there was little uncertainty around delineating this area as a contaminated area. 

**Figure 4:**
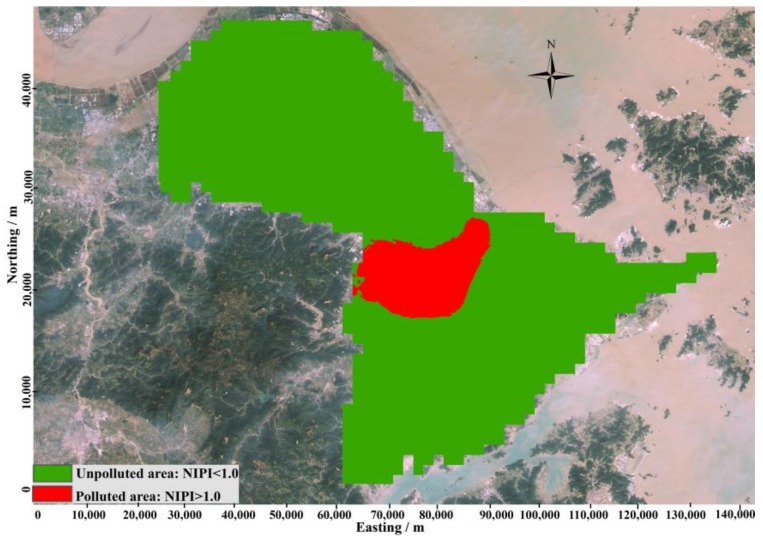
Delineation of composite heavy metal pollution according to the spatial distribution of Nemerow integrated pollution index (NIPI).

The spatial distribution of NIPI in study area was shown in Figure 4 which was got by OK. The red areas represent areas with NIPIs larger than 1.0, and are regarded as polluted areas. The green areas represent areas with NIPIs less than 1.0, and are regarded as unpolluted areas. As revealed by Figure 4, the polluted areas are mainly located in the central region of the study area, and almost overlapped with an area with high probability of being an integrative polluted area, shown in Figure 3. It indicated the central region of study area was polluted by heavy metals. Since this region was the core urban area, anthropogenic activities made main contribution to heavy metals pollution in these areas.

**Figure 5:**
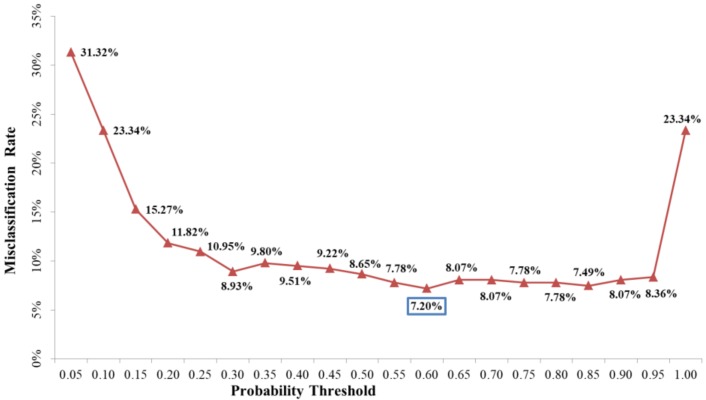
Misclassification rate vs the probability thresholds of composite heavy metal pollution risk.

**Figure 6:**
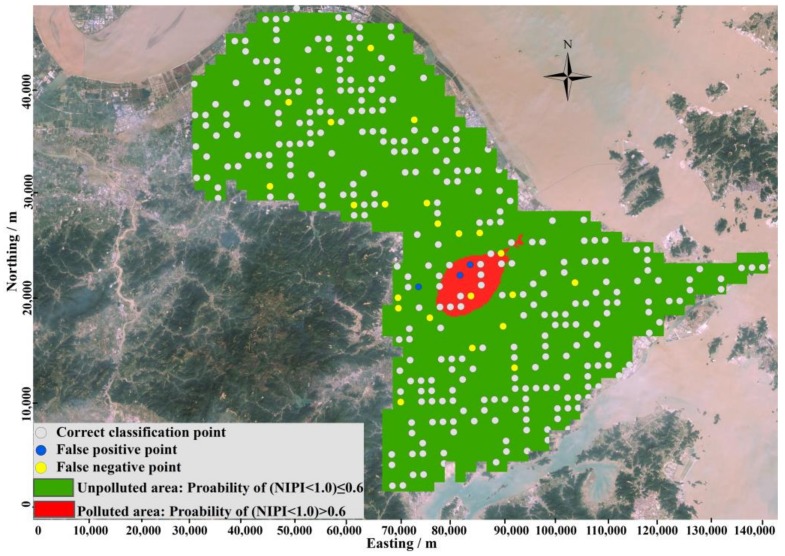
Delineation of composite heavy metal pollution according to pollution probability.

The threshold probability that was used to define polluted and unpolluted areas is critical for delineating contamination. To obtain the optimal probability for delineating pollution, a plot of the percentage of locations misclassified versus the probability threshold was generated (as shown in Figure 5). When the threshold probability is close to 1.00, the misclassification rate will be close to the proportion of integrative polluted samples with NIPI larger than 1.0, and more and more potential polluted samples would be classified as unpolluted samples. Therefore, when threshold probability increased from 0.95 to 1.00, the misclassification rate was sharply increased from 8.36% to 23.34%, and vice versa. The probability value at which a misclassification was minimal was 0.60. In addition, the misclassification rate was 7.20%. This threshold was finally chosen to classify areas as polluted and unpolluted lands as shown in Figure 6. According to the threshold level, polluted areas are mainly distributed in the central part of study area, which is consisted with the result revealed by Figure 3 and Figure 4.

Then, the result of pollution scope delineation based on probability threshold was used to assess the validation subset. After that, the assessment based on probability threshold delineation was compared with the assessment based on NIPI to define the accuracy of pollution scope delineation. A false positive error occurs when unpolluted samples are classified as polluted, and a false negative error occurs when polluted samples are classified as unpolluted. 

As represented by Figure 6, false positive errors occurred rarely, and are mainly located on polluted regions. These samples are mainly located at the contaminated area or the boundary between the contaminated area and the uncontaminated area. When using IK to obtain spatial pollution probabilities, the probabilities are affected by the surrounding polluted samples, and contamination probabilities in these areas will be higher than their actual values. However, false negative errors mainly occurred in isolation in many areas of the study area. This result mainly occurred because the NIPIs in these locations were high compared with surrounding locations, and when using IK to obtain spatial pollution probabilities that are affected by surrounding unpolluted areas, contamination probabilities in these areas will be lower than their actual values. These locations were also easily ignored, and thus, the threats to the health of citizens were also ignored. So, enough attention is needed to focus on these locations to provide potential and hidden health caused by soil heavy metals pollution in these locations. 

The misclassification rate of contamination delineation method is based on spatial distribution of NIPI (Figure 4), and the contamination delineation method based on uncertainty probability (Figure 6) were calculated and compared, as represented in Table 4. When pollution is defined based on the spatial pollution of NIPI (as shown in Figure 4), the validation samples with false negative errors totaled 17, which is 4.90% of the total study area. In addition, samples with false positive errors totaled 17, which accounted for 4.90% of the total study area. Overall, the accuracy of classification was 90.20%.

When delineate pollution based on uncertainty of heavy metals pollution as shown in Figure 6, the validation samples with false negative errors totaled 22, which is 6.34% of the total study area. In addition, samples with false positive errors totaled 3, which accounted for 0.86% of the total study area. Overall, the accuracy of classification was 92.8%. This result indicated that this method can delineate pollution with very high accuracy, and the accuracy is higher than the pollution delineation method based on spatial distribution of NIPI obtained by OK. In addition, this method was also very easy to implement. 

## 4. Conclusions

Surface soil contamination by eight studied heavy metals was evaluated using PI and NIPI with a set of 1040 soil samples in a coastal industrial city in the YRD of China. The spatial distribution of heavy metals was assessed by OK. In addition, IK was used to investigate the spatial uncertainty of the probability of pollution and to delineate soil heavy metal contamination. The results showed that the mean concentrations of all the heavy metals in the samples were less than the mean concentrations in the national secondary standard. The mean PI of Cr, Pb, Hg, Cd, AS, Cu, Zn and Ni were 0.24, 0.13, 0.54, 0.31, 0.24, 0.33, 0.40 and 0.48, respectively, all of which were at safe levels. The mean value of the NIPI was 0.59, which was also at a safe level.

The central region was the core urban area in the study area. Heavy metals were most accumulated in this area. This region also had large probabilities of composite heavy metal pollution, with pollution probabilities even higher than 0.8. A concern is that this region also has the highest population density. Thus, measures should be taken to hinder the heavy metal accumulation that occurs there.

After validation with the validation subset samples, 0.60 was selected as the optimal probability to use to define contamination locations. According to the spatial distribution of uncertainty probability of composite heavy metals contamination (Figure 3), polluted zones were mainly distributed in the central part of the study area. The sample proportion of false negative errors was 6.34% compared with 4.90% calculated from the pollution delineation method based on OK, while the percentage of samples with false positive errors was 0.86% compared with 4.90% when using pollution delineation method based on OK. The accuracy of classification was 92.80% compared with 90.2% using pollution delineation method based on OK, which indicated that this method could delineate pollution regions with very higher and satisfied accuracy. 

However, to more precisely delineate the polluted areas, more attention needs to be paid to the following issues:

(1) The available content of heavy metals should be used to replace the total concentrations of heavy metals to get a conclusion which is closer to reality.
(2) Other factors that control metal bioavailability, such as chemical partitioning, which in turn is affected by soil chemical properties, should also be considered.
(3) In this study, sample density was still sparse, and sampling density needs to be improved to obtain a higher resolution map.
(4) In this study, the ratio of the sample number of validation subset and calibration subset is 1:2, and the validation subset was randomly extracted from samples. However, the proportion of sample number of validation subset and calibration subset and the spatial pattern of validation subset may have a certain effect on the choice of optimum threshold probability which is used to define pollution sites.
(5) Contamination in soils cannot be adequate, and thresholds based on local variability should be used for properly assessing heavy metals contamination, which cannot archived by IK.

## Figures and Tables

**Table 1:** Descriptive statistics of heavy metal concentrations in the research area (mg/kg).

Items	As	Cd	Cr	Cu	Hg	Ni	Pb	Zn
Sample numbers	1040	1040	1040	1040	1040	1040	1040	1040
Mean	6.55	0.19	61.84	33.87	0.27	23.85	39.86	99.60
Std	2.27	0.37	23.92	31.50	0.33	11.12	18.97	40.60
Min	1.80	0.01	6.50	1.00	0.02	4.00	16.00	31.50
Max	29.10	11.76	262.70	685.40	3.42	131.99	313.00	749.90
CV (%)	34.61	195.13	38.68	92.99	121.82	46.61	47.59	40.77
Background value [48]	5.75	0.161	56.1	23.1	0.076	20.7	36.2	86.6
Data distribution	Log ND ^†^	Log ND	Log ND	Log ND	Log ND	Log ND	Log ND	Log ND

^†^ ND is the abbreviation of normal distribution.

**Table 2:** Descriptive statistics of the single heavy metal pollution index (PI) of heavy metals in the study area.

Items	Cr	Pb	Hg	Cd	As	Cu	Zn	Ni
Sample numbers	1040	1040	1040	1040	1040	1040	1040	1040
Mean value	0.24	0.13	0.54	0.31	0.24	0.33	0.40	0.48
Std	0.10	0.06	0.66	0.62	0.08	0.31	0.16	0.22
Min	0.03	0.05	0.03	0.02	0.06	0.01	0.13	0.08
Max	1.31	1.04	6.84	19.60	0.97	6.85	3.00	2.64
CV (%)	41.58	47.67	121.86	195.24	34.63	92.91	40.73	46.62

**Table 3:** Parameters of the semivariogram models of different heavy metals.

Elements	Model Types	C_0_	C	A_0_ (m)	R^2^	RSS	C_0_/(C)	Data Distribution
Cr	Spherical	0.007	0.040	31,100	0.973	3.69 × 10^−5^	17.7%	Log ND ^†^
Pb	Exponential	0.009	0.003	54,500	0.978	8.24 × 10^−6^	26.0%	Log ND ^†^
Cd	Exponential	0.014	0.028	11,700	0.703	6.06 × 10^−5^	49.8%	Log ND ^†^
Hg	Spherical	0.032	0.187	38,200	0.966	1.24 × 10^−3^	17.1%	Log ND ^†^
As	Spherical	0.011	0.023	42,400	0.987	2.69 × 10^−6^	46.7%	Log ND ^†^
Cu	Spherical	0.024	0.084	17,000	0.904	2.82 × 10^−4^	28.9%	Log ND ^†^
Zn	Exponential	0.007	0.020	38,100	0.978	3.03 × 10^−6^	36.0%	Log ND ^†^
Ni	Spherical	0.005	0.050	26,300	0.900	2.43 × 10^−4^	10.6%	Log ND ^†^

^†^ ND is the abbreviation of normal distribution.

**Table 4:** Statistics of the delineated area (total validation sample number = 347).

Items	Classification Based on Uncertainty Probability of NIPI > 1		Classification Based on Spatial Distribution of NIPI	
	Sample Number	Proportion	Sample Number	Proportion
False positive errors	3	0.86%	17	4.90%
False negative errors	22	6.34%	17	4.90%
Correct	322	92.80%	313	90.20%
